# Investigating mechanisms of laser pulse-induced reflectivity modulations in photoacoustic remote sensing with a 10 million frames-per-second camera

**DOI:** 10.1038/s41598-023-30831-5

**Published:** 2023-03-07

**Authors:** Nathaniel J. M. Haven, Matthew T. Martell, Haoyang Li, James D. Hogan, Roger J. Zemp

**Affiliations:** 1 Electrical and Computer Engineering Department, University of Alberta, Edmonton, T6G 2R3 Canada; 2 Mechanical Engineering Department, University of Alberta, Edmonton, T6G 2R3 Canada

**Keywords:** Biomedical engineering, Imaging and sensing

## Abstract

Photoacoustic remote sensing has been recently developed as an all-optical imaging modality capable of imaging a variety of endogenous contrast agents label-free. Initially predicted laser pulse-induced refractive index perturbation-based interrogation beam reflectivity modulations have been found to be orders of magnitude smaller than those typically observed experimentally. In this report we utilize a 10 million frames-per-second camera to further investigate these predicted reflectivity modulations, while also exploring other potential mechanisms of laser pulse-induced reflectivity modulations. Laser-induced motion is demonstrated both laterally for gold wires suspended and submerged in air and water, respectively, and carbon fibers submerged in water, and axial motion is observed in gold wires submerged in a depth gradient of intralipid solution. This laser-induced sample motion is anticipated to cause reflectivity modulations local to the interrogation beam profile in microscopy set-ups. Non-motion-based maximum intensity modulations of 3% are also observed in gold wires submerged in water, indicating the presence of the originally predicted reflectivity modulations. Overall, these observations are important as they provide a widefield view of laser-pulse interactions unavailable in previous point scanning-based photoacoustic remote sensing microscopy configurations, where observed mechanisms occur on time-scales orders of magnitude faster than equivalent field of view point scanning capabilities.

## Introduction

Photoacoustic microscopy (PAM) generates absorption-contrast imagery by tuning an excitation wavelength to match chromophore absorption peaks and using an ultrasound transducer to detect acoustic waves generated via the photoacoustic effect following excitation pulse absorption^1^. Recently, we developed photoacoustic remote sensing (PARS) microscopy, an all-optical non-contact version of PAM, demonstrating high sensitivity to a wide variety of endogenous chromophores^2–5^ and ready integration into existing technologies due to its all-optical architecture^6–10^. In comparison to PAM, PARS microscopy utilizes an additional interrogation beam to probe reflectivity modulations rather than directly detecting generated acoustic waves using an ultrasound transducer. Without a priori knowledge of the underlying mechanism, early work strove to explain the intensity modulations observed when pulsed laser light was absorbed. Initially, a model was described in which reflectivity modulations were attributed to elasto-optic refractive index perturbations arising due to photoacoustic initial pressure transients following excitation pulse absorption^2^. However, in practice predicted reflectivity modulations have been found to be orders of magnitude smaller than those typically observed experimentally, and other recent work has suggested the presence of laser-induced cavitation, which may affect reflectivity^11^. In this report, a 10 million frames-per-second (fps) camera is utilized to investigate other potential mechanisms of laser pulse-induced reflectivity modulations, with the hypothesis that these modulations may be additionally attributed to laser-induced sample motion or surrounding media displacement local to the detection beam profile.

A literature review finds several different mechanisms in which laser-induced sample motion is generated. Phase-sensitive optical coherence tomography (OCT) has recently shown that thermoelastic expansion following 5-ns pulsed excitation absorption can generate deformations with axial displacements on the order of several hundred nanometers for a $\sim$ 255 mJ/cm$^2$ pulse fluence, with the magnitude of deformation being excitation wavelength-dependent^12,13^. Nanosecond pulsed laser-induced bubble interactions with micro-particles have also been observed using high-speed cameras at up to 540,000 fps, describing particle dynamics in relation to proximity to the bubble center. In closest proximity, rapid ejection of the particle occurred along with the formation of a cavity behind the particle. Lower proximity resulted in particle repulsion that accompanied bubble expansion, followed by attraction of the particle during bubble collapse^14,15^. Since laser pulse absorption can result in pressure wave generation via the photoacoustic effect in the form of ultrasound and/or shockwaves, induced cavitation^16^ may also interact with particles, with observations made at up to 1 Mfps^17,18^. Additionally, microjets may form at boundaries^19^ or even following shockwave-induced bubble collapse^20^, exerting forces on nearby targets. These small volume liquid jets have even been utilized in microfluidic cell sorting applications to move cells between channels^21^. Laser-induced ablation recoil momentum has been demonstrated to cause sample motion through ejection of ablated material opposite to the laser irradiation direction^22^. Using cantilever dynamics and nanosecond pulsed laser fluences of up to 1400 J/cm$^2$, as much as 12 $\upmu$m normal displacement was observed with recoil momentum responding approximately linearly with laser fluence^23^. For immobile samples, 70-ns pulsed Er:YAG laser irradiation with up to 5.4 J/cm$^2$ pulse fluence generated laser tissue ablation consisting of a series of material ejection processes followed by recoil-induced material expulsion and mechanical deformation of non-ablated material^24^. Optical tweezers can be used in the manipulation of small dielectric particles exploiting photon gradient forces, with radiation pressure also exerting a force in the direction of beam propagation. These forces however are very small, typically being on the order of piconewtons^25^. Alternatively, acoustic radiation force, describing the force exerted on targets by acoustic waves, has been shown to induce micron order displacements in tissues^26^. This effect has also been explored in the form of photoacoustic radiation force on microbubbles^27^. Photoacoustic/photothermal tweezers have recently demonstrated target manipulation within absorbing medium with theoretical forces on the order of nanonewtons, with the caveat that the technique relies on the generation of acoustic and thermal gradients which may induce many physical phenomena responsible for observed motion, requiring further exploration^28^. This example in particular is very compelling, displaying the complexity and range of mechanisms that may follow laser pulse absorption. Together with refractive index perturbations, one or more of these mechanisms may also contribute to observed reflectivity modulations in PARS microscopy explaining the magnitude of reflectivity modulation measurements.

To investigate the presence of sample motion in PARS microscopy, a detection scheme utilizing widefield parallelized 10 Mfps readout was combined with laser pulse excitation fluence levels comparable to those reported in previous PARS microscopy set-ups. Compared to previous experiments which were only capable of observing a singular post pulse excitation frame at a synchronized 10 fps readout^29^, the orders-of-magnitude higher frame rate in this work enables observation of motion dynamics on the sub-microsecond scale. Additionally, we demonstrate that the magnitude of observed intensity modulations arising potentially due to refractive index-based perturbations are significantly weaker than the magnitude of observed motion in samples, providing further evidence for the hypothesis of motion-based reflectivity modulations. The manuscript is laid out as follows: details are provided pertaining to system characterization; results of non-motion-based intensity modulations are described; lastly, observations of laser-induced sample motion in air and in liquid are shown with discussion as to the potential underlying mechanisms responsible.

## Results

### System characterization

**Figure 1:**
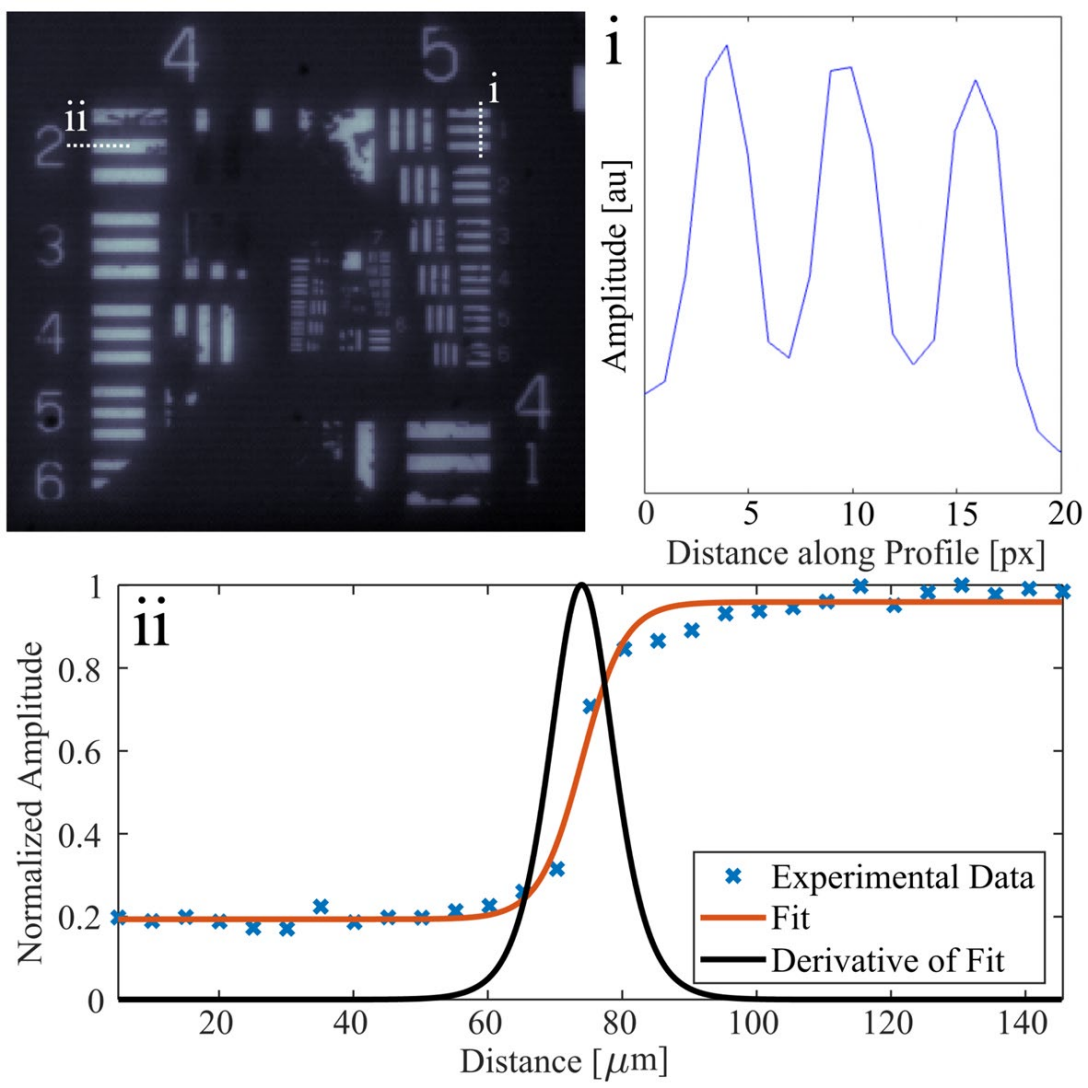
Image of a 1951 USAF resolution test target with extraction of: (**i**) line-pair width profile used to determine effective pixel size, and (**ii**) edge-spread measurement across a target edge for lateral resolution characterization.

First we characterized the lateral resolution of our system. A 1951 USAF resolution test target was imaged with our system and the denoted line-pair widths were used to determine an effective pixel size of 5 $\upmu$m as shown in Fig. 1i. Using the effective pixel size to determine image dimensions, an edge-spread function was measured from the target, allowing us to calculate a full-width at half-maximum (FWHM) lateral resolution of 12 $\upmu$m as shown in Fig. 1ii, fit using a logistic function. Note that using widefield excitation illumination, the depth resolution of our system will be determined by the interrogation beam depth of field, and has been calculated to be on the order of $\sim$62 $\upmu$m. Using the provided camera lens (K2 DistaMax, CF-2) and our system objective (Mitutoyo, Plan Apo 10X), a system magnification of $\sim$ 1 was achieved. Theoretical calculations for the camera resolution agree with experimental values, where we would expect a resolution of $\sim$ 11.5 $\upmu$m, accounting for the Nyquist limit.

### Detecting non-motion-based intensity modulations

**Figure 2:**
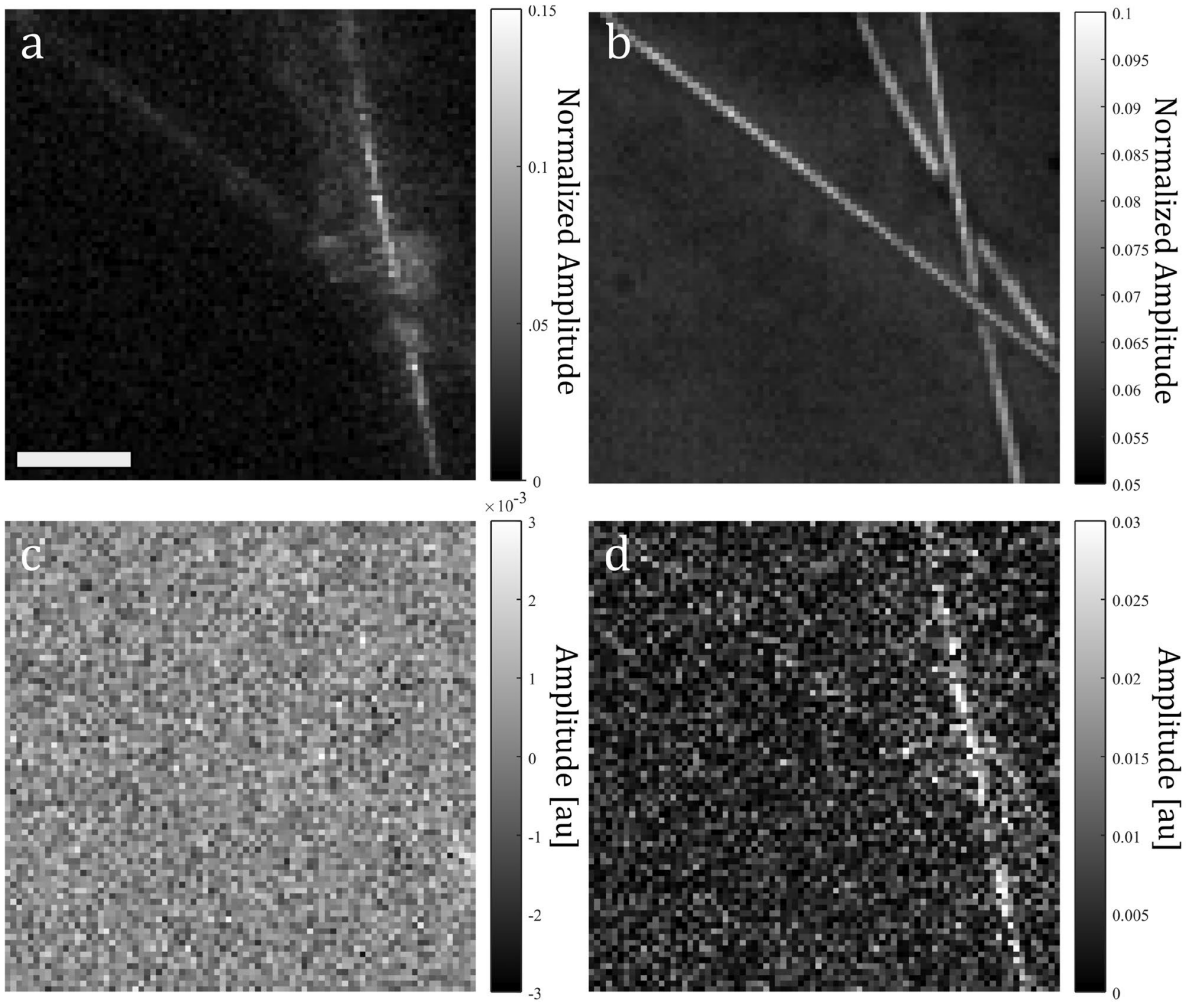
Results from three sequential acquisitions imaging 25 $\upmu$m diameter gold wires covered with a layer of water and coverslip: (**a**) excitation frame from acquisition A; (**b**) mean of the pre-excitation frames from acquisition B; (**c**) difference between the mean of the pre-excitation frames in acquisition B and mean of frames in acquisition C; (**d**) difference between the excitation frame in acquisition B and the output in (**b**) followed by a difference between this result and the output in (**a**). Scale bar: 100 $\upmu$m.

The system was next utilized to explore widefield parallelized camera-based detection of intensity modulations following laser pulse excitation. Despite best efforts, laser pulse excitation leakage in the form of diffuse scattering from the target into the camera sensor was unavoidable due to the strong angle of incidence dependence of the notch filter used. To address this leakage, three acquisitions were taken sequentially of 25 $\upmu$m diameter gold wires covered with a layer of water and coverslip: acquisition A containing an excitation laser pulse event with no interrogation source; acquisition B containing an excitation laser pulse event with interrogation source; and acquisition C contained only the interrogation source. Fig. 2a shows the excitation frame for acquisition A (n = 53) showing the unavoidable leakage signal, where the amplitude has been normalized to the dynamic range of the camera. As a reference, Fig. 2b shows the mean of the pre-excitation frames (n = 1–52) in acquisition B. To ensure no motion-based modulations between acquisitions, Fig. 2c shows the difference between the image shown in Fig. 2b and the mean frame of acquisition C, with no apparent difference. To isolate the intensity modulation, a difference between the excitation frame in acquisition B (n = 53) and the mean of its pre-excitation frames (Fig. 2b) was taken followed by a difference between this resulting image and the excitation frame in acquisition A (Fig. 2a), with the result shown in Fig. 2d, where a maximum intensity modulation of $\sim$ 3 $\%$ is observed in some regions. A signal-to-noise ratio (SNR) of $\sim$ 15 dB was measured across the image, calculated by taking the ratio of the mean value of a representative signal to the standard deviation of the background noise. This low SNR suggests an imaging sensitivity not currently suitable for biological tissue imaging, however this could be improved in future work by introducing a flash lamp as the detection source, or transitioning to a transmission-mode set-up, where both of these changes would significantly improve dynamic range utilization of the camera sensor, thus improving sensitivity. These results represent the first report of intensity modulations detected using a camera in a PARS system, indicating the presence of non motion-based reflectivity modulations first predicted in the original PARS report^2^.

### Detecting laser-induced motion in targets suspended in air

**Figure 3:**
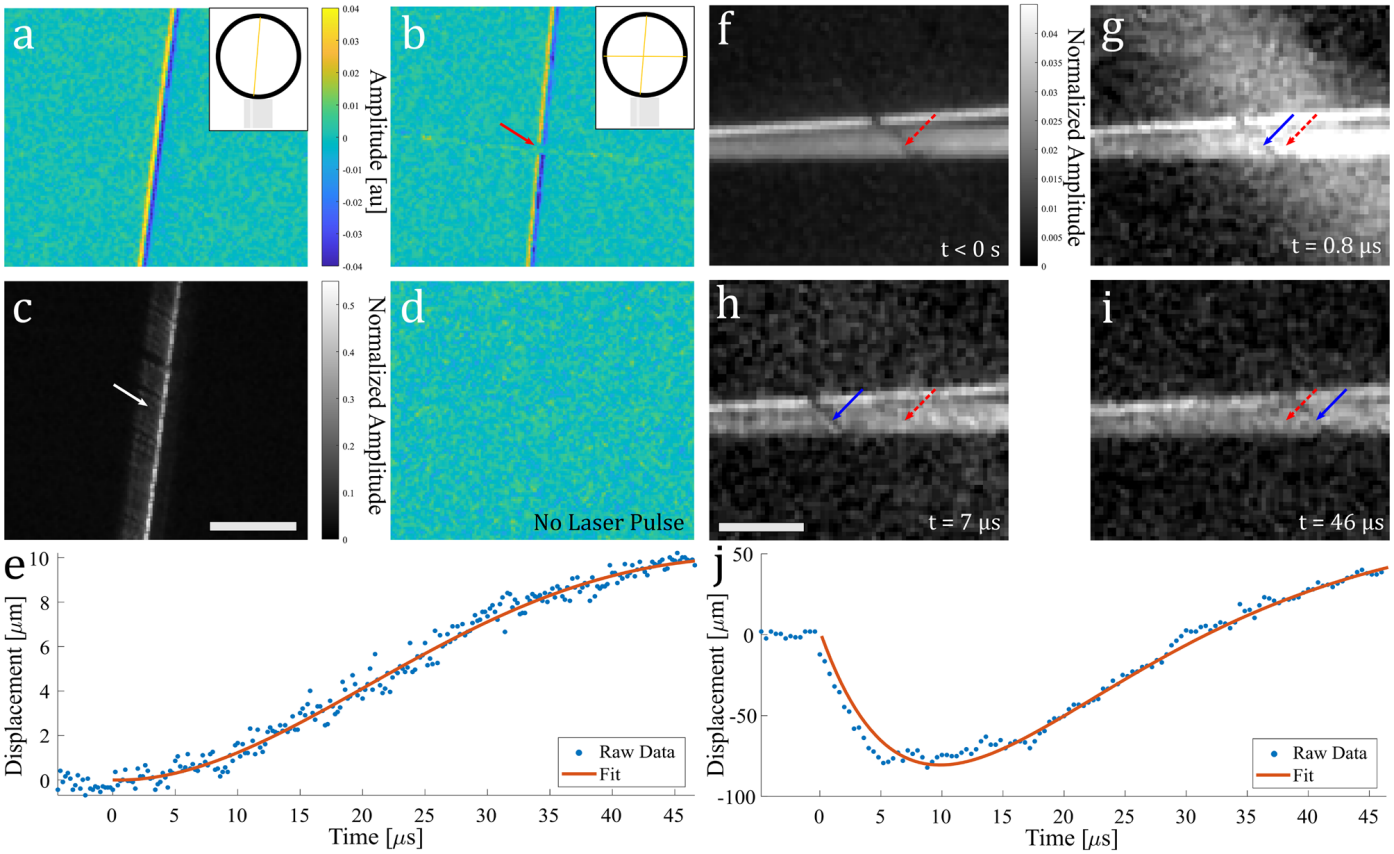
(**a**) Single and (**b**) crossed 25 $\upmu$m diameter gold wire in air and in tension last frame and mean of pre-excitation frames difference image; (**c**) excitation frame for (**a**); (**d**) first and last frame difference image with no excitation pulse; scale bar: 200 $\upmu$m. (**e**) Wire displacement plot against time for the motion shown in (**a**). (**f**) pre-excitation frame of a 7 $\upmu$m diameter carbon fiber target diagonally suspended in air and in tension in front of a pair of 50 $\upmu$m diameter aluminum and 25 $\upmu$m diameter gold wires, with the red dashed arrow indicating pre-excitation fiber location; (**g**)–(**i**) fiber location displacement over time with the solid blue arrow indicating the current frame fiber location; scale bar: 100 $\upmu$m. (**j**) Fiber displacement plot against time for the motion shown in (**g**)–(**i**).

To investigate potential laser-induced sample motion in air, a vertical 25 $\upmu$m diameter gold wire target was suspended in air and in tension as described in Fig. 3a inset. In the absence of any excitation laser pulse, comparing the final frame of the acquisition (n = 256) to the first frame (n = 1) by taking a difference image as shown in Fig. 3d, an absence of signal is clearly shown indicating insensitivity to external background stimuli over the duration of the acquisition. In contrast, in the presence of an excitation laser pulse, comparing the final frame of the acquisition (n = 256) to the mean of the pre-excitation pulse frames by taking a difference image, it is clearly seen in Fig. 3a that movement of the wire has occurred in the direction of the incident excitation laser beam (incident from the right side), where positive values corresponds to final wire position, and negative values corresponds to initial wire position. The full motion dynamics can be viewed in Supplementary information Video S1. A plot of wire displacement against time for an arbitrary y location is shown in Fig. 3e following rotation of the wire using bicubic interpolation, and using spline interpolation to provide a finer sampled estimate of the wire’s position. The data was fit using an exponentially decaying sinusoid function. The maximum velocity reached by the wire is measured to be $\sim$ 0.3 m/s. Repeating the same test for a crossed gold wire phantom, as illustrated in the inset of Fig. 3b, no significant lateral motion is observed on the horizontal wire indicated by the red solid arrow. It should be noted that due to a large depth of field, we do not expect axial motion to be observable in this configuration. Figure 3c displays the excitation frame, note that since the image is not saturated, the streaking highlighted by the white solid arrow may suggest emission of ablated material from the gold wire rather than camera pixel blooming. To test for the presence of ablation, an additional experiment was conducted where the same experimental set-up and parameters were utilized to repeatedly irradiate a 25 $\upmu$m diameter gold wire suspended in air and in tension. The results are shown in Supplementary information Fig. S2, where there is a clear change in the wire following repeated irradiation ($\sim$ 2 s continuous) with eventual breakage of the wire when the laser irradiation duration is long enough ($\sim$ 30 s in this experiment).

We next imaged a target consisting of a 7 $\upmu$m diameter carbon fiber diagonally suspended in air and in tension in front of a pair of 50 $\upmu$m diameter aluminum and 25 $\upmu$m diameter gold wires. The metal wires were used as a reflective background for the carbon fiber which can be seen as a dark line on top of the wires. As demonstrated in Fig. 3b, a horizontal arrangement is not expected to result in any observable motion, allowing carbon fiber motion to be analyzed independently of the metal wires. Figure 3f shows the mean of the pre-excitation pulse frames, where the silhouette of the carbon fiber is more visible. The dashed red arrows and solid blue arrows correspond to the fiber’s position pre-excitation pulse and its current position in the frame, respectively. Post excitation pulse Fig. 3g,h show the carbon fiber’s displacement to the left (away from the incident pulse direction). After 7 $\upmu$s however, the fiber begins to move in the opposite direction, eventually passing its original position as seen in Fig. 3i. The full motion dynamics can be viewed in Supplementary information Video S2. Averaging every pair of sequential frames and plotting the fiber’s displacement along an arbitrary y location over time and using an exponentially decaying sinusoid function fit we get the trajectory shown in Fig. 3j, where no appreciable motion is observed until the excitation pulse at t = 0. In contrast to Fig. 3e, the displacement trajectory shows a much higher measured maximum velocity of $\sim$ 21.6 m/s, and the presence of restoring forces that result in a response following that of an underdamped oscillator. Additional displacement trajectory plots for carbon fiber phantoms can be seen in Supplementary information Fig. S3a, where the fluence was varied to test the displacement response. In these results, as the fluence is increased the maximum displacement also increases, with a linear relationship shown in Supplementary information Fig. S3b. It is also interesting to note that in this experiment, as the fluence was increased past a certain threshold, the fiber underwent a plastic response wherein it no longer returned back to its original position. Note that in sample preparation the tensile load was limited to ensure the wires/fibers did not break when attached to the outside holding frame, thus we may not expect these wires to completely settle back to their initial positions. Figure 3e also displays an eventual deceleration which may indicate the presence of similar restoring forces. However, due to a restricted total observation time during the acquisition we cannot be certain. Note that the bright cloud-like region to the right of the fiber in Fig. 3g may also be emitted material following ablation of the fiber.

### Detecting laser-induced motion in targets submerged in liquid

**Figure 4:**
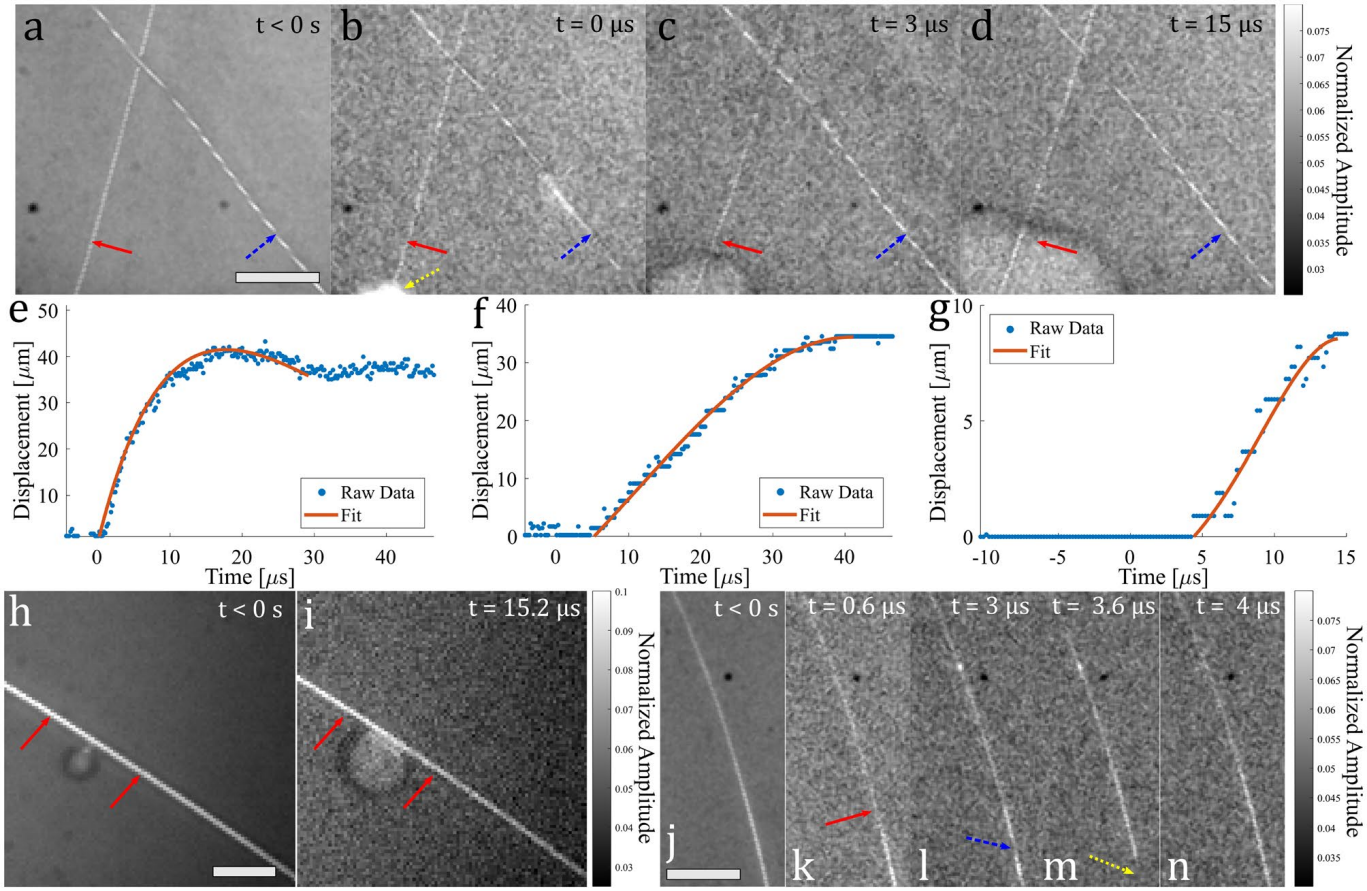
(**b**)–(**d**) Motion in crossed 25 $\upmu$m diameter gold wires submerged in a thin layer of distilled water and covered with a coverslip following laser-induced bubble generation, with (**a**) showing the mean of pre-excitation frames, scale bar: 200 $\upmu$m. In (**a**)–(**d**) Solid red and dashed blue arrows indicate the initial position of the left and right wires, respectively. (**e**), (**f**) Wire displacement against time for both left and right wires in (**a**)–(**d**), respectively. (**h**) mean of pre-excitation frames for a 25 $\upmu$m diameter gold wire touching a pre-existing bubble and (**i**) motion following pulse excitation, with red solid arrows indicating initial positions, scale bar: 100 $\upmu$m. (**g**) Wire displacement against time for (**h**), (**i**). (**j**) Mean of pre-excitation frames for a 25 $\upmu$m diameter gold wire submerged in a layer of intralipid scattering media, with the top being more submerged than the bottom; (**k**)–(**n**) motion following pulse excitation with arrows indicating regions on interest, scale bar: 200 $\upmu$m.

Next, to explore potential laser-induced sample motion in liquid media, 25 $\upmu$m diameter gold wires were submerged in a thin layer of distilled water and covered with a coverslip to follow previously reported sample preparation used in PARS microscopy experiments^10^. It should be noted that when submerged in water there appeared to be an absence of target ablation possibly due to better stress propagation in water compared to air, this likely explains the reliance on this type of sample preparation in PARS microscopy experiments. In the presence of water, laser-induced bubble formation was observed with bubble dissipation occurring as early as 10 $\upmu$s post-excitation. In the majority of cases, complete dissipation of bubbles occurred prior to subsequent acquisitions, which was verified in sequentially taken acquisitions as can be seen in Supplementary information Fig. S1. Additionally, Supplementary information Fig. S1 shows that bubble nucleation locations and quantity of bubbles varies across acquisitions, this is likely due to the probabilistic nature of cavitation effects which is more pronounced when using widefield excitation illumination. Figure 4a shows the mean of the pre-excitation pulse frames with solid red and dashed blue arrows indicating the initial positions of each respective wire. After laser pulse excitation a bubble is induced in the area indicated by the yellow dotted arrow in Fig. 4b, with rapid motion occurring in the wire contained within this bubble as the bubble expands as shown in Fig. 4c. Eventually, motion is also observed in the further wire as seen in Fig. 4d. The full motion dynamics can be viewed in Supplementary information Video S3. Wire displacement plots in time for both the left and right wires from an arbitrary y location using an exponentially-decaying sinusoid function fit are shown in Fig. 4e,f, respectively. From Fig. 4e motion in the wire occurs immediately following the laser excitation pulse, with a measured maximum velocity of $\sim$ 10 m/s. At around t = 15 $\upmu$s post-excitation, some indication of restoring forces are observed when the wire begins to return to its original position, until plateauing occurs at a fixed wire displacement. In contrast, Fig. 4f shows a delayed motion response following pulse excitation, with a maximum velocity of $\sim$ 1.35 m/s reached. Assuming that bubble nucleation is responsible for the motion in the right wire, an interaction with the right wire occurring at a distance of $\sim$ 460 $\upmu$m away from the left bubble nucleation site, following a delay of $\sim$ 5 $\upmu$s, suggests an interaction velocity of $\sim$ 92 m/s. In the presence of pre-existing bubbles, bubble expansion is triggered by laser pulse excitation, as shown in Fig. 4i, where red solid arrows indicate the wire’s position pre-excitation, with Fig. 4h showing the mean of the pre-excitation pulse frames. This also demonstrates how a bubble in contact with the wire pushes the wire as it expands. The full motion dynamics can be viewed in Supplementary information Video S4. The trajectory of this motion for an arbitrary y location is displayed in Fig. 4g, fit using an exponentially-decaying sinusoid function, and indicates that there is a $\sim$ 5 $\upmu$s delay before motion is observed, with a maximum velocity of $\sim$ 1 m/s.

Lastly, in an attempt to observe axial motion within the system’s large depth of field, we imaged a target consisting of a 25 $\upmu$m diameter gold wire submerged in a layer of intralipid scattering media, where the wire’s submerged depth was set up to vary from top to bottom by at most one coverslip thickness (130–170 $\upmu$m). The mean of the pre-excitation frames, shown in Fig. 4j shows the depth gradient profile across the gold wire, with wire depth increasing at the top of the image. Following laser pulse excitation there are clear changes in intensity at the bottom of the wire as indicated by the red solid arrow, blue dashed arrow, and yellow dotted arrow in Figs. 4k–m respectively, followed by a return to a pre-excitation state after t = 4 $\upmu$s as shown in Fig. 4n. With no observed lateral motion in this example, these intensity changes could be due to axial motion of the wire in and out of the intralipid solution, or intralipid media displacement. The full motion dynamics can be viewed in Supplementary information Video S5.

## Discussion

In this work, we report the observation of laser-induced motion in a variety of phantoms using widefield parallelized 10 Mfps readout. Targets suspended in tension in air indicated the potential emission of ablated material, which may suggest a laser-induced ablation recoil momentum source to explain the motion. In carbon fibers, restoring forces were also observed in the displacement trajectory demonstrating a similar response to that of an underdamped oscillator. We hypothesize that this tension-induced restoring force may be more prevalent in focused laser pulse experiments where local structural forces should be stronger relative to induced localized motion. Targets submerged in water indicated motion due to laser-induced bubble formation, with the magnitude and velocity of displacement being dependent on proximity to the bubbles or whether bubbles were pre-existing. In closer proximity to the bubble nucleation site, these results also indicated the presence of restoring forces on the phantoms. Nonlinear displacement trajectories observed suggest that we should expect nonlinear reflectivity modulation responses in a point-based PARS microscopy set-up for dominant motion-based mechanisms. Additionally, axial motion was explored using scattering media to generate a depth gradient across the target, with results indicating an intensity change along the target following pulse excitation, which suggests axial motion of the sample or displacement of the surrounding scattering media. Targets submerged in water showing no bubble formation post pulse-excitation were not observed to exhibit any in-plane lateral motion. The absence of apparent laser-induced ablation recoil momentum in these cases may be due to better stress propagation in water compared to air. Weak intensity modulations observed following pulse leakage removal are believed to be due to the refractive index-based reflectivity perturbation mechanism previously reported.

In conclusion, we hypothesize that these observed motion responses, both lateral and axial, may additionally contribute to reflectivity modulations observed in PARS microscopy, with induced sample motion local to the detection beam profile generating a localized reflectivity change. These high-speed camera-based observations are important as they provide a widefield view of excitation pulse interactions unavailable in previous point scanning-based PARS microscopy configurations, where observed mechanisms occur on time-scales orders of magnitude faster than equivalent field of view point scanning capabilities. Future work should investigate the presence of this observed motion in a point scanning-based PARS microscopy system using methods capable of detecting localized sample displacement with high sensitivity, such as phase-contrast OCT^30^ and laser Doppler vibrometry^31^, although it should be noted that these methods would only be able to measure axial motion. Moreover, work should also explore the presence of other potential mechanisms such as photoacoustic radiation force, thermal expansion, and cavitation. In particular, in immersion environments, even if bubble nucleation is absent, motion may result from photoacoustic radiation pressure being created on one side of the target where absorption is strong, but not on the other side of the target where there is minimal fluence. Better understanding the underlying mechanisms in PARS may allow more effective detection of laser-induced changes, better informing system design characteristics and sample preparation for improved PARS microscopy imaging. Additionally, the potential for decoupling signals according to their respective source mechanisms may provide further information not currently available in PARS microscopy images.

## Methods

**Figure 5:**
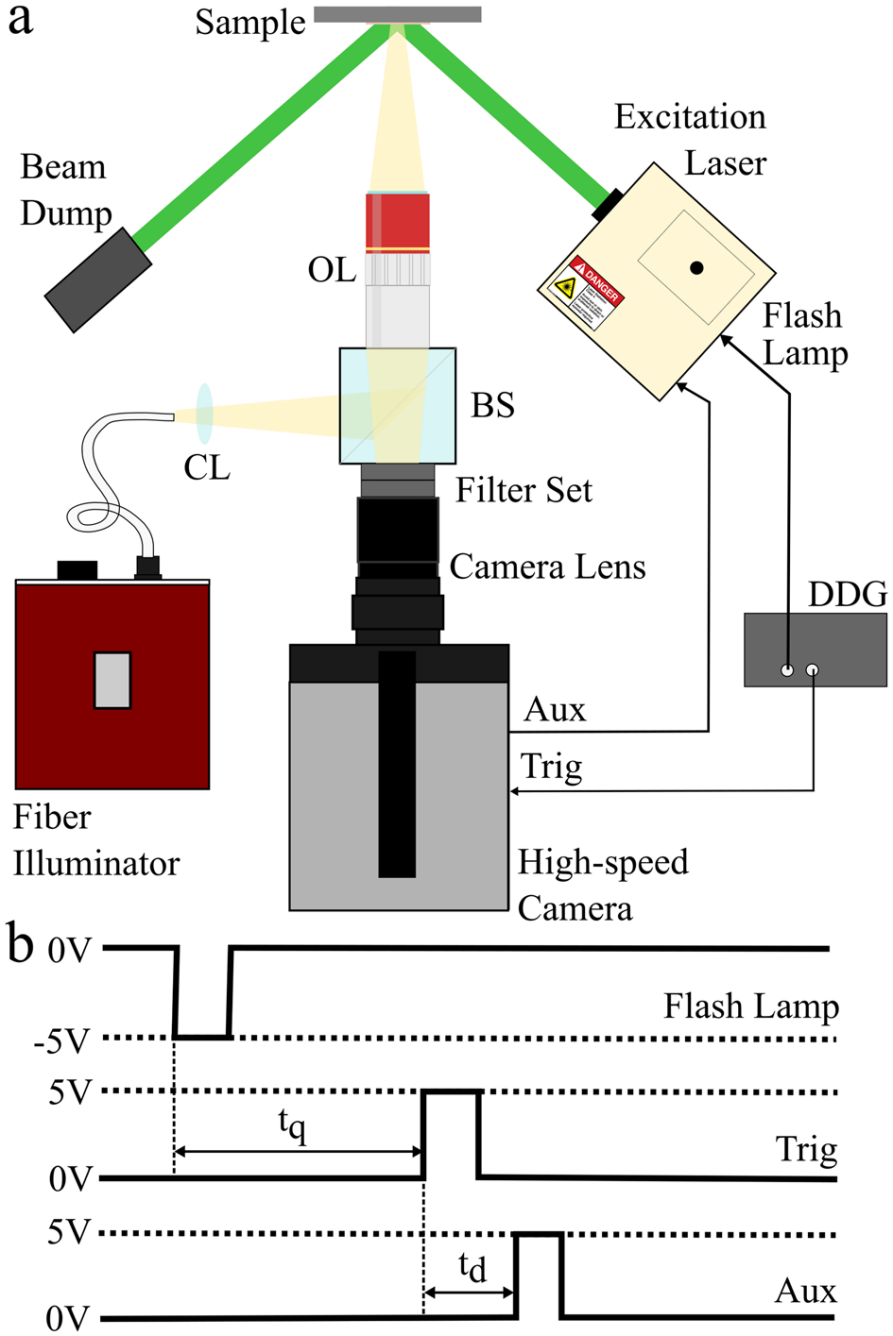
(**a**) Experimental set-up. OL, objective lens; DDG, digital delay generator; CL, collimating lens; BS, beamsplitter. (**b**) Timing diagram for synchronizing camera capture with excitation laser pulsing, top: signal to initiate flash lamp laser pumping; middle: Q-switch time-delayed signal to initiate camera acquisition; bottom: nanosecond-delayed signal to trigger a laser pulse.

Figure 5a shows our system diagram, where a 532 nm nanosecond pulsed Nd:YAG 10 Hz excitation laser (Continuum, Surelite SLIII-10) obliquely irradiated our sample before being collected by a beam dump. Our interrogation beam originated from a fiber-coupled illuminator (Thorlabs, OSL2) fitted with a high-power bulb variant (Thorlabs, OSL2B2), and a collimating lens (Thorlabs, LA1289). This beam was split using a 50:50 beamsplitter cube (Thorlabs, CCM1-BS013) and focused onto the sample using a 0.26 numerical aperture (NA) objective lens (Mitutoyo, Plan Apo 10X). The back-reflected interrogation light then travelled back through the 50:50 beamsplitter cube and passed through a notch filter (Thorlabs, NF533-17) and shortpass filter (Thorlabs, FESH1000). The notch filter was intended to remove stray 532 nm excitation light diffusely scattered from the sample, and the shortpass filter used to reject any residual 1064 nm leakage from the 532 nm laser pump. The remaining interrogation light was then collected into the camera lens (K2 DistaMax, CF-2), which focused the light onto the high-speed camera sensor (Shimadzu, HyperVision HPV-X2).

In order to ensure that we were able to capture a signal immediately following the excitation event, the following synchronization set-up was used, with a timing diagram shown in Fig. 5b: a digital delay generator (SRS, DG645) internal trigger output was used to continuously stimulate flash-lamp pumping of the excitation laser at 10 Hz. A second output from the delay generator, a t = $t_q$ delayed signal with respect to the delay generator internal trigger rate, then triggered the camera to start an acquisition. The camera was preset to acquire a certain number of pre-trigger frames, and upon being triggered, sent a signal to pulse the excitation laser with a delay t = $t_d$ with respect to the camera trigger event. This last variable delay allows temporal shifting of the laser pulse within the camera exposure cycle.

In comparison to our previously reported point-based scanning PARS microscopy, the excitation beam in this set-up is both unfocused and has oblique incidence on the sample. With point-based scanning PARS, in order to image 7 $\upmu$m diameter carbon fibers, typically a pulse energy of 1 nJ at 532 nm using a 0.40 NA objective is required^2^, resulting in a fluence of 177.6 mJ/cm$^2$. To match this fluence using oblique irradiation, assuming an effective 1.41 cm beam diameter on the sample, and an incidence angle of 45$^\circ$, we required a pulse energy of 276.4 mJ. This pulse energy is well within the capability of the excitation laser, corresponding to a Q-switch delay of $\sim$ 330 $\upmu$s.

The high-speed camera uses a FTCMOS2 image sensor with 10-bit monochrome output and is capable of recording at 10 Mfps, with 50 ns exposure time, and 5 Mfps with 110 ns exposure time. When operating at 5 Mfps, the full 100,000 pixels can be used, however, when operated at 10 Mfps, half the pixels are used in a zigzag lattice pixel array. Based on previous PARS microscopy modulation observations, a 10-bit dynamic range is anticipated to be sufficient to detect intensity modulations, previously predicted to be on the order of $10^{-4}-10^{-2}$ in magnitude^2^. Additionally, taking into consideration the Nyquist limit, 10 Mfps camera capture should provide a detection bandwidth of 5 MHz. Compared to the typical photodiode bandwidth of 75 MHz used in previous point-based PARS microscopy systems^32^, we expect a loss in detectable frequency content which could result in aliasing in the case of detecting high-frequency modulations greater than 5 MHz. However, for the purposes of capturing motion, these high-speed cameras have been used without issue to image frames of high-speed events involving projectiles moving at $\sim$ 1078 m/s, which should far exceed what we expect to detect^33^.

## Supplementary information

Supplementary Information.

Supplementary Video S1.

Supplementary Video S2.

Supplementary Video S3.

Supplementary Video S4.

Supplementary Video S5.

## Data Availability

Relevant data and code will be made available upon reasonable request by the corresponding author.
