# Supplementary material for: Investigating mechanisms of laser pulse-induced reflectivity modulations in photoacoustic remote sensing with a 10 million frames-per-second camera
Source: Sci Rep. 2023 Mar 7;13:3751. doi: 10.1038/s41598-023-30831-5 (PMC9992668; doi:10.1038/s41598-023-30831-5)
Supplement: Supplementary file 1 — Supplementary Information. [file 41598_2023_30831_MOESM1_ESM.pdf]

Supplementary Information

Nathaniel J. M. Haven[1], Matthew T. Martell[1], Haoyang Li[2], James D. Hogan[2], and Roger J. Zemp[1,*]

[1]University of Alberta, Electrical and Computer Engineering Department, Edmonton, T6G 2R3, Canada
[2]University of Alberta, Mechanical Engineering Department, Edmonton, T6G 2R3, Canada
[*]rzemp@ualberta.ca

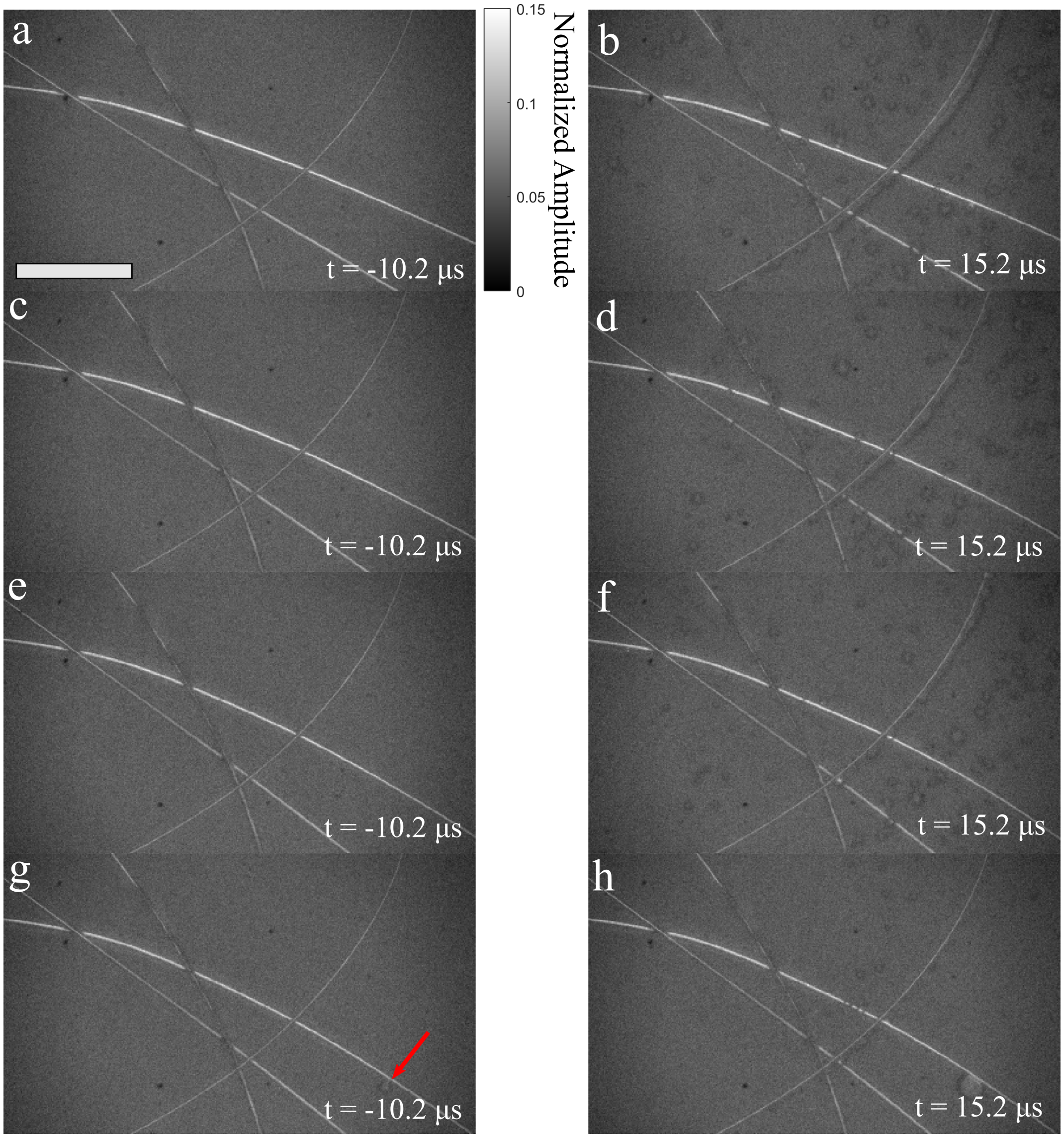

Figure S1. First and last frame of 4 sequential acquisitions showing laser-induced bubble formation in 25 μm diameter gold wires submerged in water during the acquisition and dissipation prior to the following acquisition. a-b, c-d, e-f, and g-h correspond to acquisition 1, 2, 3, and 4, respectively, each taken as soon as possible after the previous acquisition. The red arrow in g indicates the presence of a non-dissipated bubble. Scale bar: 500 μm.

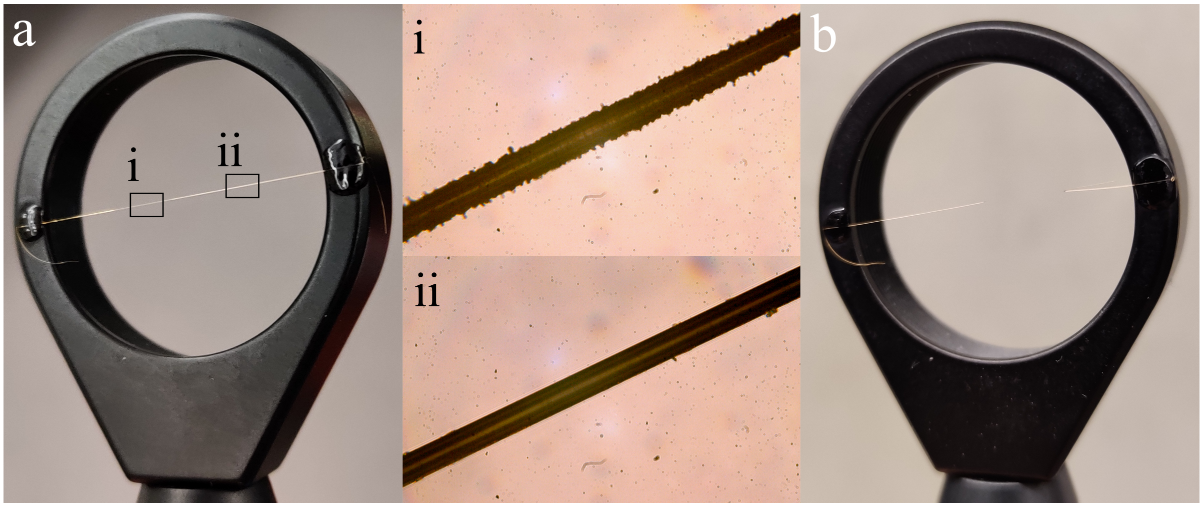

Figure S2. a) 25 μm diameter gold wire suspended in air, where i) and ii) indicate regions exposed or not exposed to repeated laser irradiation, respectively. i)/ii) 40X brightfield microscopy zoom-in images of each respective region in a). b) State of the gold wire following ~30s of laser irradiation.

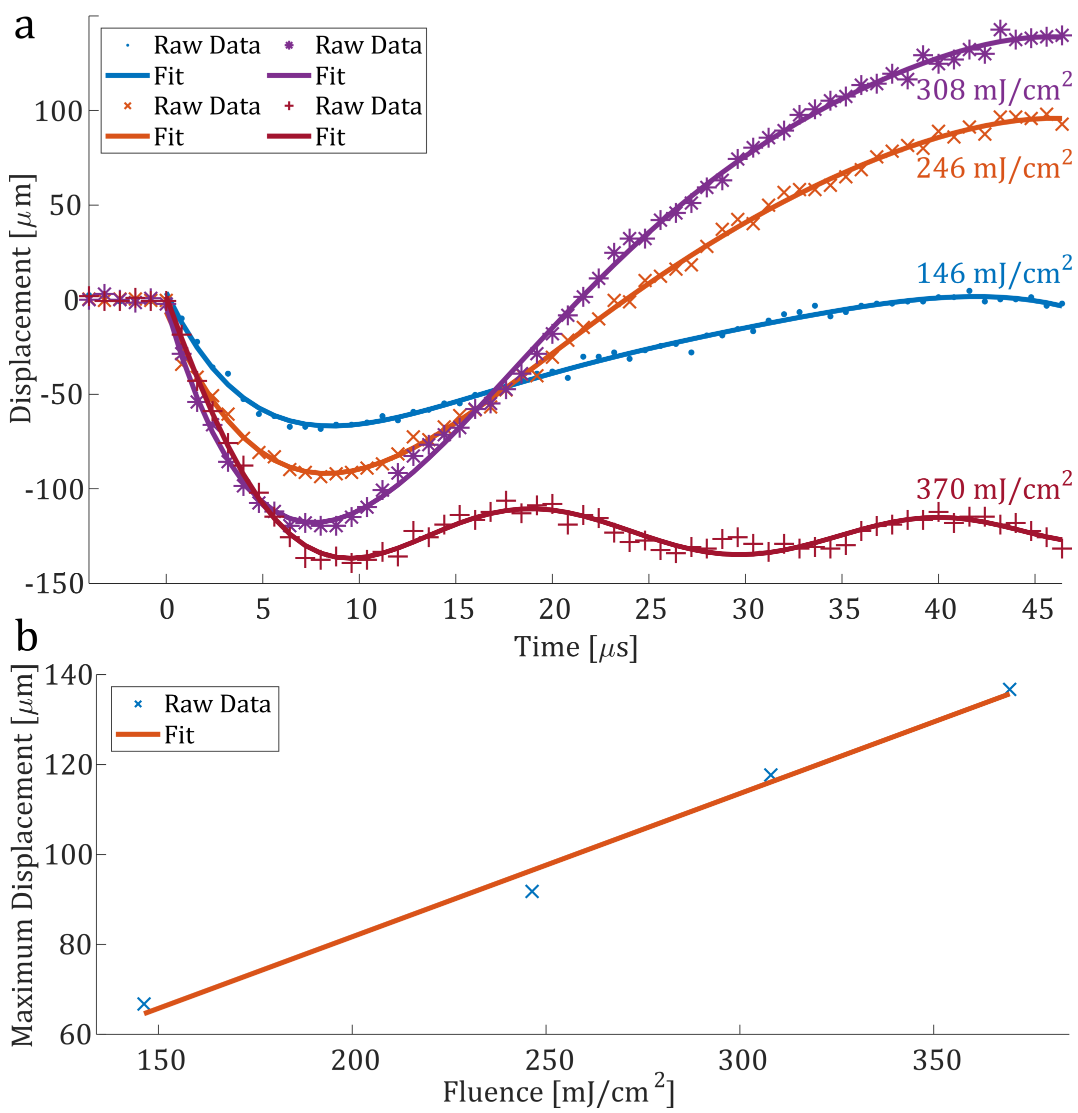

Figure S3. a) 7 μm diameter carbon fiber displacement plot against time for different incident laser fluence values. b) Plot of the absolute maximum displacement in a) against incident laser fluence.

Video S1. Video of the normalized difference image between each frame and the mean of pre-excitation frames for a 25 μm diameter gold wire in air and in tension. Scale bar: 500 μm.

Video S2. Video of motion in a 7 μm diameter carbon fiber target diagonally suspended in air and in tension in front of a pair of 50 μm diameter aluminum and 25 μm diameter gold wires, with the red solid arrow indicating the pre-excitation laser pulse fiber location. Scale bar: 100 μm.

Video S3. Video of motion following laser-induced bubble generation in a crossed 25 μm diameter gold wire phantom submerged in a thin layer of distilled water and covered with a coverslip. Solid red and blue arrows indicate the initial position of the left and right wires, respectively. Scale bar: 200 μm.

Video S4. Video of motion in a 25 μm diameter gold wire submerged in a thin layer of distilled water and covered with a coverslip touching a pre-existing bubble. Red solid arrows indicate initial positions of the gold wire. Scale bar: 100 μm.

Video S5. Video of motion in a 25 μm diameter gold wire submerged in a layer of intralipid scattering media, with the top being more submerged than the bottom. Scale bar: 500 μm.
